# The Prognostic Role of Plasma Epstein-Barr Virus DNA Levels in the Middle of Intensity Modulated Radiation Therapy to Guide Cisplatin Dose Recommendation in Concurrent Chemoradiation Therapy in Patients With Locally Advanced Nasopharyngeal Carcinoma: A Large Cohort Study

**DOI:** 10.1016/j.adro.2022.100908

**Published:** 2022-02-03

**Authors:** Zhen-Chong Yang, Chao-Chao Du, Li-Ting Liu, Yu-Jing Liang, Lin-Quan Tang, Qiu-Yan Chen, Hai-Qiang Mai, Shan-Shan Guo

**Affiliations:** Sun Yat-sen University Cancer Center, State Key Laboratory of Oncology in South China, Collaborative Innovation Center for Cancer Medicine, Guangdong Key Laboratory of Nasopharyngeal Carcinoma Diagnosis and Therapy, Guangzhou, China; and Department of Nasopharyngeal Carcinoma, Sun Yat-sen University Cancer Center, Guangzhou, China

## Abstract

**Purpose:**

Our purpose was to investigate the prognostic role of plasma Epstein-Barr virus (EBV) DNA levels in the middle of intensity modulated radiation therapy (IMRT).

**Methods and Materials:**

In total, 1881 patients with stage III-IVa tumors were included. The overall survival (OS) and progression-free survival (PFS) were calculated using the Kaplan-Meier method, and the differences were compared using the log-rank test. Receiver operating characteristic curve analysis was performed to analyze the diagnostic value of EBV DNA levels for tumor progression or death. Multivariate analyses using the Cox model were used to evaluate potential prognostic factors.

**Results:**

The positive predict value and negative predict value of plasma EBV DNA > 0 copies/mL in the middle of IMRT in predicting nasopharyngeal carcinoma progression was 37.4% and 85.5%, respectively. In patients with plasma EBV DNA level = 0 copies/mL, no significant differences in OS were observed between patients treated with 200 mg/m² cisplatin and those treated with >200 mg/m² cisplatin (5-year OS, 94.9% vs 94.4%; PFS, 81.5% vs 87.6%). However, those treated with >200 mg/m² cisplatin had higher PFS. In patients with plasma EBV DNA level > 0 copies/mL, patients treated with >200 mg/m² cisplatin displayed a favorable 5-year OS (84.6% vs 73.9%) and PFS (72.3% vs 54.8%) compared with those treated with 200 mg/m² cisplatin. Additionally, higher incidences of grade 3 and 4 adverse events were recorded in patients treated with >200 mg/m² cisplatin than in those treated with 200 mg/m² cisplatin.

**Conclusions:**

Plasma EBV DNA > 0 copies/mL in the middle of IMRT suggests that higher doses of chemotherapy should be used. For concurrent chemoradiation therapy, >200 mg/m² cisplatin is recommended for patients with plasma EBV DNA level > 0 copies/mL in the middle of IMRT but not for patients with plasma EBV DNA level = 0 copies/mL considering the similar OS rates.

## Introduction

Nasopharyngeal carcinoma (NPC) is a unique malignancy that is endemic in South China and is associated with Epstein-Barr virus (EBV) infection in most cases.^1^^,^^2^ Normally, NPC risk stratification is mainly based on the tumor, node, metastasis (TNM) staging system. However, NPC is invariably associated with an EBV infection^3^^,^^4^ and plasma EBV DNA testing is widely known for its proven ability for tumor surveillance in patients with NPC.^5^ Particularly, pretreatment and posttreatment plasma EBV DNA levels correlate with NPC survival and progression.^6^^,^^7^ However, previous studies that have focused on plasma EBV DNA levels in the middle of intensity modulated radiation therapy (IMRT) either had a small sample size or lacked data from the high incidence areas of the Chinese mainland.^8^ Therefore, there is insufficient evidence to explain the clinical role of plasma EBV DNA levels in the middle of IMRT. Furthermore, whether plasma EBV DNA in the middle of IMRT could guide the third cycle of cisplatin in concurrent chemoRT (CCRT) remains unclear.

The National Comprehensive Cancer Network guidelines recommend CCRT with 100 mg/m² cisplatin (DDP) every 3 weeks for patients with stage II-IVa NPC, based on the findings of several prospective randomized trials and meta-analyses.^9^, ^10^, ^11^ However, some patients with NPC could not tolerate CCRT with >200 mg/m² DDP because of gastrointestinal reactions, ototoxicity, and neurotoxicity caused by DDP.^12^ Multiple randomized controlled clinical trials of chemoRT have shown that only 52% to 63% of patients with locally advanced NPC could complete chemotherapy with >200 mg/m² DDP in the same period owing to toxicity and side effects caused by chemotherapy.^13^^,^^14^ Although CCRT improves the overall survival (OS) rate of NPC, there is currently no evidence that >200 mg/m² DDP is more beneficial than 200 mg/m² of DDP.^15^^,^^16^

In this study, we used plasma EBV DNA levels in the middle of IMRT to classify patients into different risk groups and evaluated the efficacy of 200 mg/m² DDP versus >200 mg/m² DDP in patients from different risk groups. We hope that the data generated in this study may provide an additional dimension for risk stratification and individualized therapy for patients with NPC.

## Methods and Materials

### Patients

**Fig. 1:**
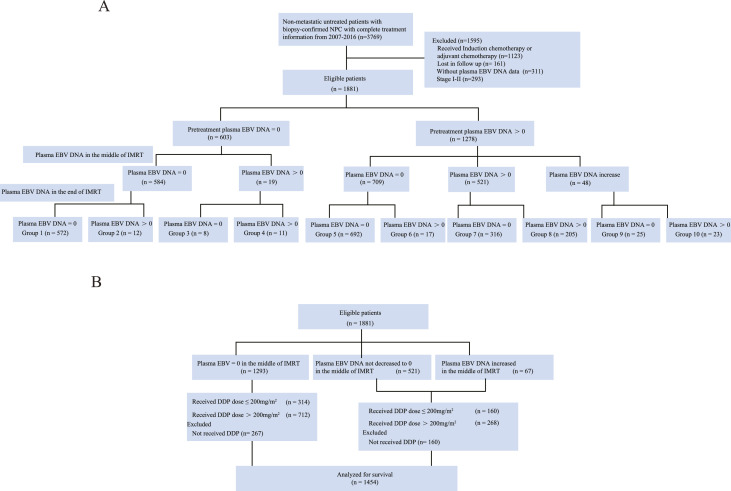
Consort diagram of the study populations. Plasma EBV DNA levels are present in copies/mL. Abbreviations: DDP = cisplatin; EBV = Epstein-Barr virus; IMRT = intensity modulated radiation therapy; NPC = nasopharyngeal carcinoma.

From October 2007 to October 2016, 3769 nonmetastatic and untreated patients with biopsy-confirmed NPC were identified at Sun Yat-sen University Cancer Center. The eligibility criteria were as follows: (1) age ≥ 18 years; (2) stage III-IVa NPC according to the 8th edition of the American Joint Committee on Cancer staging system; (3) score of 0 or 1 as per the Eastern Cooperative Oncology Group performance status grade; (4) treatment with IMRT; (5) complete data of pretreatment, middle of treatment, and posttreatment plasma EBV DNA levels; (6) adequate hematological, liver, and renal function parameters. Patients undergoing induction chemotherapy or adjuvant chemotherapy, those who were pregnant or lactating, and those who had distant metastasis or a prior malignancy were excluded from the study. In total, 1881 eligible patients were included for analysis (Table 1 and Fig. 1). This study was approved by the clinical research and ethics committee of our institute. This work was carried out in accordance with The Code of Ethics of the World Medical Association (Declaration of Helsinki) for experiments involving humans.

**Table 1:** Baseline characteristics in the study population

Variable, n (%)	Total (n = 1881)	EBV DNA = 0 in the middle of IMRT (n = 1293)	EBV DNA decrease in the middle of IMRT (n = 521)	EBV DNA increase in the middle of IMRT (n = 67)	P value	χ
Age (y)					.088	4.863
≤50	1223 (65.0)	861 (70.4)	323 (26.4)	39 (3.2)		
>50	658 (35.0)	432 (65.7)	198 (30.1)	28 (4.3)		
Sex					.173	0.917
Male	1367 (72.7)	937 (68.5)	382 (27.9)	48 (3.5)		
Female	514 (27.3)	356 (69.3)	139 (27.0)	19 (3.7)		
ECOG					<.001	19.777
0	492 (26.2)	366 (74.4)	101 (20.5)	25 (5.1)		
1	1389 (73.8)	927 (66.7)	420 (30.2)	42 (3.0)		
Smoking					.365	2.013
No	1186 (63.1)	829 (69.9)	316 (26.6)	41 (3.5)		
Yes	695 (36.9)	464 (66.8)	205 (29.5)	26 (3.7)		
Family history of NPC					.631	0.921
No	1667 (88.6)	1149 (68.9)	461 (27.7)	51 (3.4)		
Yes	214 (11.4)	144 (67.3)	60 (28.0)	10 (4.7)		
T stage					<.001	25.930
1	52 (2.8)	36 (69.2)	14 (26.9)	2 (3.8)		
2	219 (11.6)	138 (62.8)	76 (34.9)	5 (2.3)		
3	1240 (66.0)	893 (72.0)	299 (24.1)	48 (3.9)		
4	370 (19.7)	226 (61.1)	132 (35.7)	12 (3.2)		
N stage					<.001	87.216
0	244 (13.0)	204 (83.6)	31 (12.7)	9 (3.7)		
1	739 (39.3)	554 (75.0)	163 (22.1)	22 (3.0)		
2	770 (40.9)	474 (61.5)	267 (34.7)	29 (3.8)		
3	128 (6.8)	61 (47.7)	60 (46.9)	7 (5.5)		
Total stage					<.001	30.903
III	1400 (74.4)	1009 (72.1)	341 (24.2)	50 (3.6)		
IVa	481 (25.6)	284 (59.0)	180 (37.4)	17 (3.5)		
Pretreatment EBV DNA levels					<.001	765.103
0	603 (32.1)	584 (96.8)	0 (0.0)	19 (3.2)		
1-4000	544 (28.9)	455 (35.2)	70 (12.9)	19 (3.5)		
4001-100,000	557 (29.6)	220 (39.5)	310 (55.7)	27 (4.8)		
>100,000	177 (9.4)	34 (19.2)	141 (79.7)	2 (1.1)		
EBV DNA levels at the end of IMRT					<.001	494.218
0	1613 (85.5)	1264 (78.4)	316 (19.6)	33 (2.0)		
>0	268 (14.2)	29 (10.8)	205 (76.5)	34 (12.7)		
Chemotherapy					.032	6.686
≤200 mg/m^2^ DDP	474 (32.6)	314 (66.2)	139 (29.4)	21 (4.4)		
>200 mg/m^2^	980 (67.4)	712 (72.7)	239 (24.3)	29 (3.0)		

Abbreviations: DDP = cisplatin; EBV = Epstein-Barr virus; ECOG = Eastern Cooperative Oncology Group; IMRT = intensity modulated radiation therapy; N stage = node stage; NPC = nasopharyngeal carcinoma; T stage = tumor stage.

### Imaging examination and EBV DNA level assessment

Before treatment, all patients underwent complete physical and imaging examinations, nasopharyngoscopy, and laboratory workup, which included a complete blood count and biochemical profile. Plasma EBV DNA levels were measured using a quantitative reverse transcription polymerase chain reaction assay that targeted the BamHI-W region of the EBV genome^14^ (Sun Yat-sen University Cancer Center Molecular Diagnostics Department) at 3 time points: pretreatment, in the middle of IMRT, and at the end of IMRT (posttreatment). Samples that showed EBV DNA levels = 0 copies/mL were considered negative. Magnetic resonance imaging of the nasopharynx and neck, chest radiography, abdominal sonography, electrocardiography, and bone scanning or ^18^F-fluorodeoxyglucose positron emission tomography/computed tomography were performed for accurate disease staging.^17^ We defined the middle of IMRT as the period within 10 to 15 doses. In our department, EBV DNA samples were collected before the chemotherapy. Thus, all the blood samples in the middle of IMRT were measured before patients received 200 mg/m^2^ DDP.

### RT therapy and chemotherapy

All patients were treated with IMRT with or without concurrent chemotherapy. The IMRT regimen was designed according to previous studies, with 66 to 70 Gy/30 to 33 fractions to the primary lesions, 60 to 70 Gy/30 to 33 fractions to the involved neck fields, and 50 to 54 Gy/30 to 33 fractions of prophylactic irradiation to the neck.^18^ Further, 1454 (77.3%) patients received concurrent DDP chemotherapy. Overall, 474 (32.6%) patients received DDP dose ≤200 mg/m^2^, 980 (67.4%) received DDP dose >200 mg/m^2^, 265 received nedaplatin as concurrent chemotherapy,^19^ and 162 did not receive concurrent chemotherapy. In our cohort of the 1454 patients, 68% of the patients were given 3 weekly DDP with 100 mg/m^2^, and 32% of the patients were given weekly DDP with 40 mg/m^2^.

### Outcomes and follow-up

The primary endpoint of the study was progression-free survival (PFS), calculated from the start of the treatment to the date of the first progression, death from any cause, or patient censoring at the last follow-up. The secondary endpoint was OS, which was defined as the time from the start of the treatment to death from any cause or patient censoring at the last follow-up. Other outcomes included locoregional-free survival, which was defined as the time from the start of the treatment until locoregional recurrence or patient censoring at the last follow-up, and distant metastasis-free survival (DMFS), which was defined as the time from the start of the treatment until distant metastasis or patient censoring at the last follow-up.

After primary treatment, the patients were assessed during their clinical follow-ups (serial history, physical examinations, and nasopharyngeal endoscope evaluations) every 3 months for the first 2 years and every 6 months thereafter. Magnetic resonance imaging of the head and neck, plasma EBV DNA level measurement, radiograph of the chest, and abdomen ultrasound were performed every 6 months for the first 3 years and every 6 to 12 months thereafter. Biopsy or 2 different imaging scans (in case biopsy was not possible) were used to confirm tumor progression in patients who showed positive surveillance results. Acute toxicities were classified according to the Common Toxicity Criteria for Adverse Events version 4.0, and late RT-related toxic effects were assessed and graded based on the Radiation Therapy Oncology Group/European Organization for Research and Treatment of Cancer morbidity scoring schema.

### Statistical analysis

Statistical analyses were conducted using SPSS version 26.0 (SPSS, Chicago, IL) and R version 4.0.0 (www.r-project.org). Statistical tests were 2-sided, and *P* ≤ .05 was considered significant. χ^2^ test and Fisher's exact test were used to compare categorical variables. Kaplan-Meier method and log-rank test were used to estimate survival curves. The diagnostic value of EBV DNA level for tumor progression or death was assessed using receiver operating characteristic curve analysis. Multivariate analyses and hazard ratios (HRs) with 95% confidence intervals (CIs) were calculated using the Cox proportional hazards model. Potential prognostic factors for PFS and OS were presented by forest plots with adjusted HRs and 95% CIs.

## Results

The baseline characteristics of 1881 patients are listed in Table 1. After a median follow-up period of 68 months (interquartile range, 53-81 months), 212 patients died, 112 developed local or locoregional recurrence, and 207 exhibited distant metastases. The median value of mid-treatment EBV DNA was 0 copies/mL (range, 0-9,830,000 copies/mL)

### Risk stratification according to EBV DNA levels in the middle of IMRT

The patients were classified into 10 different risk groups according to the plasma EBV DNA levels at the start, in the middle, and at the end of IMRT (Figs. 1 and E1). The changes in plasma EBV DNA levels during IMRT are shown in Figure E2. For groups 1 to 10, the 5-year PFS values were 87.1%, 62.5%, 37.5%, 27.3%, 88.1%, 63.0%, 79.8%, 48.3%, 59.4%, and 24.5%, respectively (*P* < .001); the 5-year OS values were 95.3%, 83.3%, 60.0%, 72.7%, 95.2%, 83.7%, 92.8%, 65.7%, 82.3%, and 39.3%, respectively (*P* < .001); and the 5-year DMFS values were 93.3%, 75.0%, 70.0%, 72.7%, 94.6%, 84.8%, 90.9%, 64.5%, 86.4%, and 44.7%, respectively (*P* < .001).

However, the survival curves for these 10 groups could not be completely separated. Moreover, the risk classification based on post-IMRT plasma EBV DNA levels could not guide the usage of concurrent chemotherapy.

**Fig. 2:**
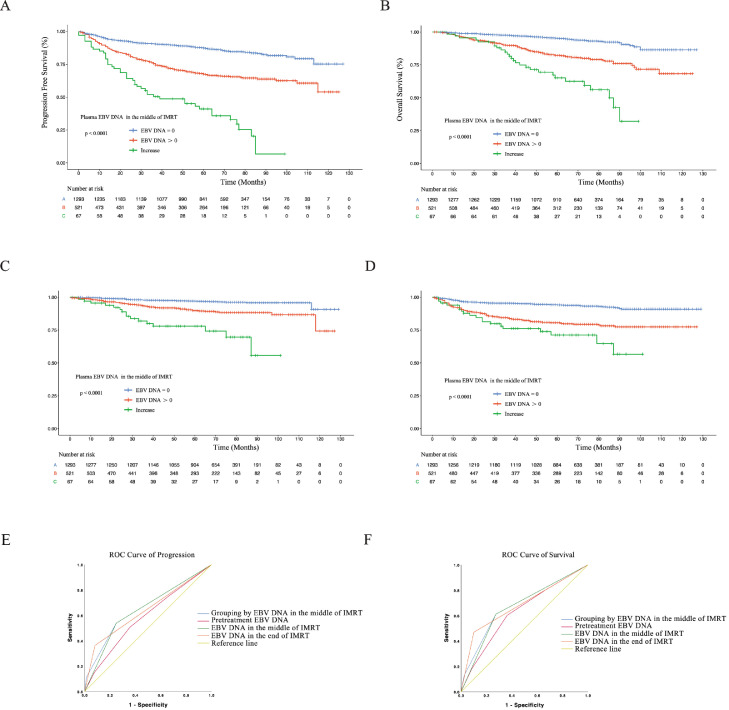
(A-D) Kaplan-Meier estimates of survival of patients with different plasma Epstein-Barr virus (EBV) DNA levels in the middle of intensity modulated radiation therapy (IMRT). (E, F) Receiver operator characteristic (ROC) curves of progression- and survival-based grouping based on different plasma EBV DNA levels.

Therefore, based on the changes in plasma EBV DNA levels during the treatment (Figs. 1A and E2), we categorized the patients into 3 different risk groups according to the plasma EBV DNA levels in the middle of IMRT (Fig. 1B) as follows: low-risk group (plasma EBV DNA level = 0 copies/mL), intermediate-risk group (plasma EBV DNA level did not decrease to 0 copies/mL), and high-risk group (plasma EBV DNA level increased). The characteristics of the patients in these risk groups are listed in Table 1. Survival curves were significantly segregated with respect to the patients in different risk groups as per the 5-year PFS (*P* < .001), OS (*P* < .001), DMFS (*P* < .001), and locoregional-free survival (*P* < .001 Fig. 2; and Table E1). The 5-year PFS was 87.1%, 67.5%, and 35.9% and OS was 95.2%, 82.0%, and 62.5% for the low-risk, intermediate-risk, and high-risk groups, respectively.

Receiver operating characteristic analyses proved that the 3 different risk groups classified based on plasma EBV DNA levels in the middle of IMRT showed higher accuracy for the prediction of progression and death than that obtained with pretreatment EBV DNA levels (progression: area under the curve [AUC] of 0.655 vs AUC of 0.586; death: AUC of 0.679 vs AUC of 0.631 Fig. 2;E, F).

**Fig. 3:**
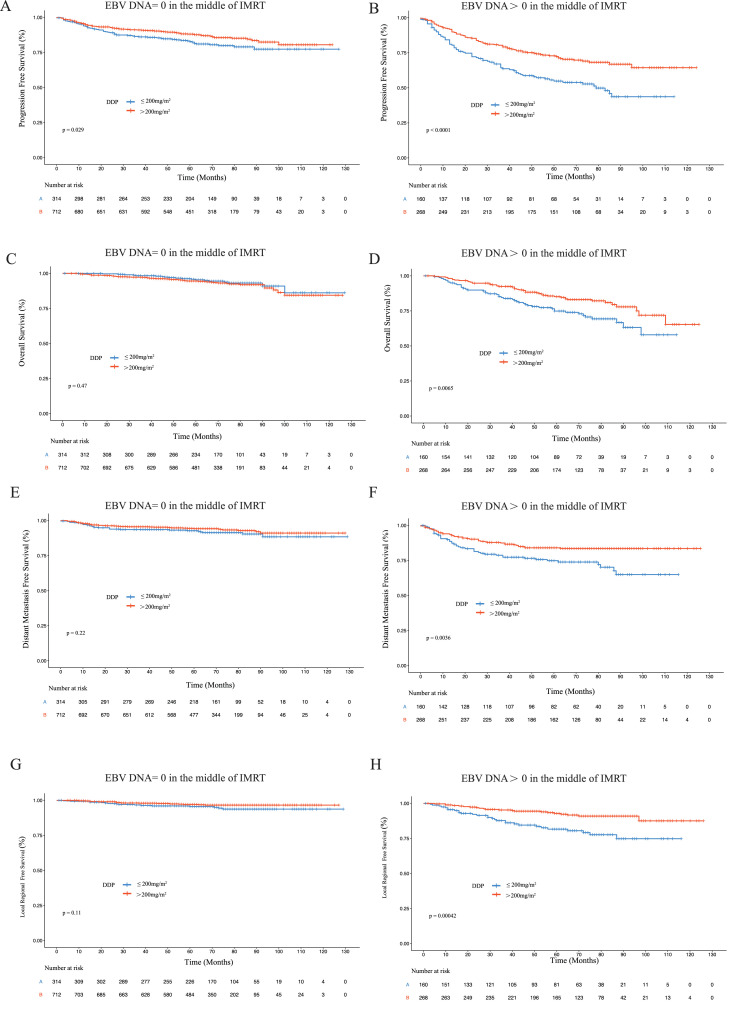
Kaplan-Meier estimates of survival of patients with different cisplatin (DDP) doses according to plasma Epstein-Barr virus (EBV) DNA levels in the middle of intensity modulated radiation therapy (IMRT).

However, the increase in plasma EBV DNA level was a rare phenomenon and was observed in only 67 patients in our cohort. Hence, for further analysis, we merged the intermediate-risk group with the high-risk group and considered plasma EBV DNA level > 0 copies/mL as the selection criteria for this merged group (Fig. 3). In this case, the specificity, sensitivity, positive predict value, and negative predict value of plasma EBV DNA in the middle of IMRT in predicting NPC progression were 75.2%, 51.6%, 37.4%, and 85.5%, respectively. For predicting NPC survival, the specificity, sensitivity, positive predict value, and negative predict value of plasma EBV DNA in the middle of IMRT were 72.6%, 61.1%, 22.0%, and 93.7%, respectively

**Fig. 4:**
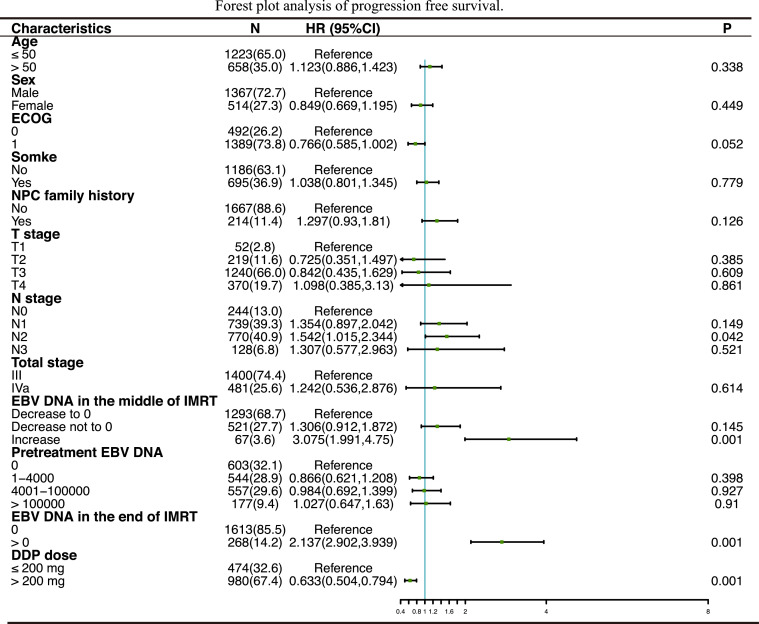
Forest plot analysis of progression-free survival. Abbreviations: CI = confidence interval; DDP = cisplatin; EBV = Epstein-Barr virus; ECOG = Eastern Cooperative Oncology Group; HR = hazard ratio; NPC = nasopharyngeal carcinoma.

We performed multivariate analyses that included sex (female or male), patient age (≤50 or >50 years), clinical tumor stage, plasma EBV DNA level, and DDP dose. Figure 4 shows that a significant protective value was present with the application of >200 mg/m^2^ DDP in the multivariate model for PFS (HR, 0.633; 95% CI, 0.504-0.794; *P* < .01) in the primary cohort including all patients. Further, the increase in plasma EBV DNA level was a risk factor for PFS (HR, 3.075; 95% CI, 1.991-4.75; *P* < .01).

### Relationship between total DDP dose and EBV DNA levels in the middle of IMRT

For patients with plasma EBV DNA level = 0 copies/mL in the middle of IMRT, no significant differences in OS rates were observed between patients treated with 200 mg/m^2^ DDP and those treated with >200 mg/m^2^ DDP. However, patients treated with 200 mg/m^2^ DDP had lower PFS rates compared with those treated with >200 mg/m^2^ (5-year OS, 94.9% vs 94.4%, *P* = .475; 5-year PFS, 81.5% vs 87.6%, *P* = .029 Fig. 3;). That is, for patients who receive DDP at 100 mg/m^2^, the third cycle of DDP may not be needed.

Nevertheless, in patients with plasma EBV DNA level > 0 copies/mL in the middle of IMRT, significantly better survival rates were observed in patients treated with >200 mg/m^2^ DDP than in those treated with 200 mg/m^2^ DDP (5-year OS, 84.6% vs 73.9%, *P* = .007; 5-year PFS, 72.3% vs 54.8%, *P* = .001 Fig. 3;).

**Fig. 5:**
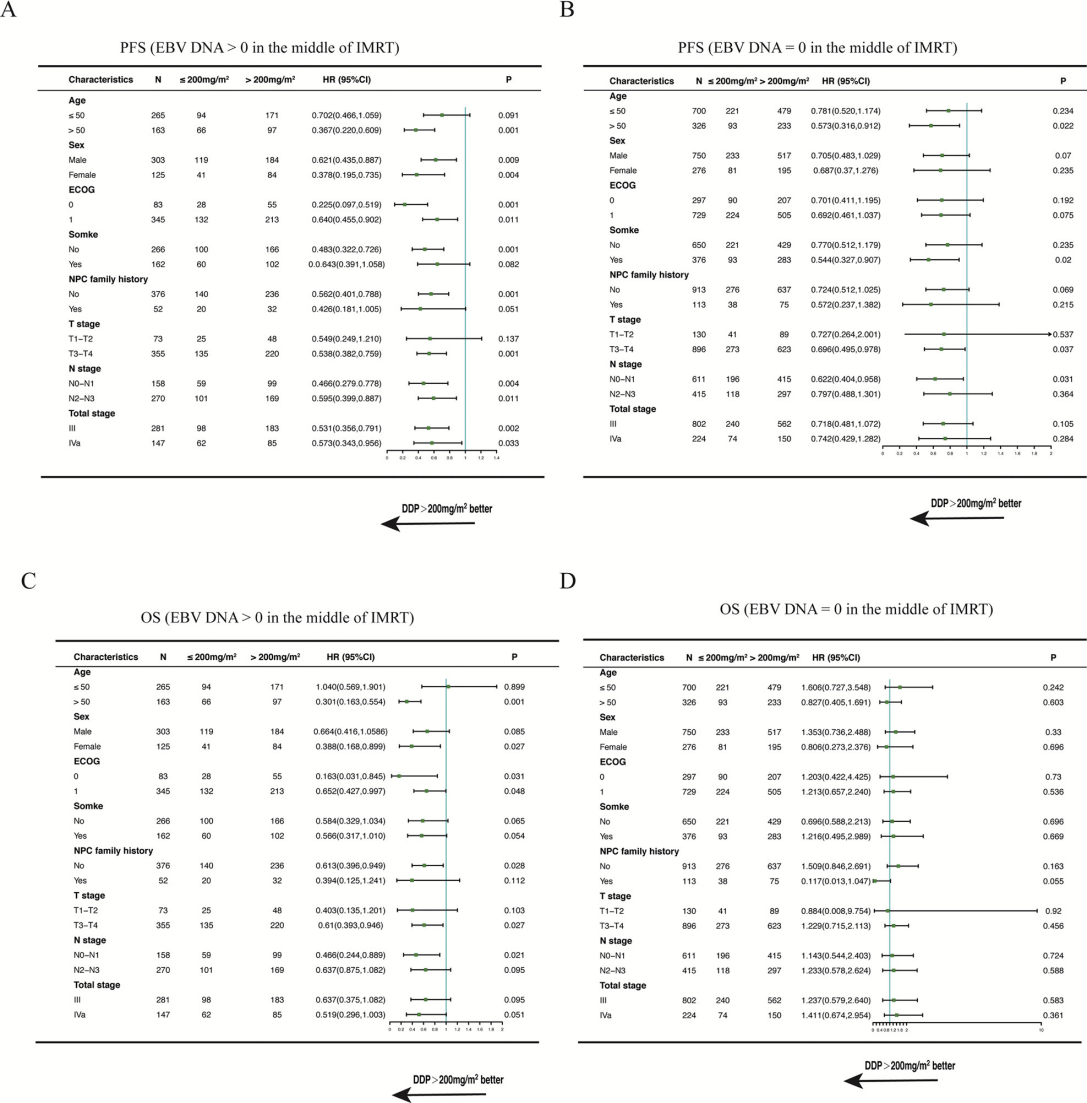
Forest plot analysis of PFS and OS in different patient subgroups. Abbreviations: CI = confidence interval; DDP = cisplatin; EBV = Epstein-Barr virus; ECOG = Eastern Cooperative Oncology Group; HR = hazard ratio; IMRT = intensity modulated radiation therapy; NPC = nasopharyngeal carcinoma; OS = overall survival; PFS = progression-free survival.

In the multivariate analysis (Fig. 5), treatment with >200 mg/m^2^ DDP was an independent prognostic factor for OS and PFS of patients with plasma EBV DNA level > 0 copies/mL in the middle of IMRT. In contrast, treatment with >200 mg/m^2^ DDP was not an independent prognostic factor for OS and PFS of patients with plasma EBV DNA level = 0 copies/mL in the middle of IMRT.

We recorded a higher frequency of grade 3 to 4 leucopenia (*P* < .05), vomiting (*P* < .05), and nausea (*P* < .05) in the DDP > 200 mg/m^2^ group than in the DDP ≤ 200 mg/m^2^ group; however, the frequencies of grade 3 to 4 late toxicities in the 2 groups were similar (Table E2).

## Discussion

To the best of our knowledge, this is the first large study to evaluate the efficacy of plasma EBV DNA levels in the middle of IMRT. Feasibility and clinical effect of postinduction chemotherapy and post-IMRT plasma EBV DNA levels are indicative of treatment outcomes.^5^ Studies focused on middle-treatment EBV DNA levels either had a small sample size or did not contain data obtained from patients in mainland China.^8^^,^^20^ Therefore, the effect of plasma EBV DNA levels in the middle of IMRT has not yet been fully studied.

In this study, we classified patients into 10 risk groups based on the levels of plasma EBV DNA during IMRT. Nevertheless, not all intergroup prognoses were significantly different. Furthermore, the plasma EBV DNA levels at the end of CCRT could not guide the concurrent chemotherapy, because patients had finished the concurrent chemoRT at the time point of the posttreatment plasma EBV DNA test. According to a previous study, plasma EBV DNA levels post-IMRT cannot guide adjuvant chemotherapy.^21^ Although pre-RT and post-RT plasma EBV DNA are good prognostic factors, they cannot guide either the application of concurrent chemotherapy or adjuvant chemotherapy. Thus, we focused on the application of the plasma EBV DNA level in the middle of IMRT treatment, which can indicate if the third cycle of DDP should be administrated.

Therefore, we emphasized the usage of plasma EBV DNA levels in the middle of IMRT because this could provide us timely guidance to set the dose during concurrent chemotherapy. Our observations also support the concept of using EBV DNA levels in the middle of IMRT for individualization of concurrent chemotherapy intensity. For patients without detectable mid-RT plasma EBV DNA, the third cycle of DDP may be spared to acquire lower toxicity. Consequently, we believe that plasma EBV DNA levels in the middle of IMRT have higher prognostic values than pretreatment EBV DNA levels. Therefore, plasma EBV DNA = 0 in the middle of IMRT was a protective prognostic factor for patients with NPC during IMRT.

In our cohort, the negative predict values of plasma EBV DNA > 0 in the middle of IMRT in predicting NPC progression and survival were 85.5%, and 93.7%, respectively. Thus, we found that plasma EBV DNA level > 0 copies/mL in the middle of IMRT was a poor prognostic factor. Although many adverse events were recorded in patients with NPC receiving >200 mg/m^2^ DDP with concurrent chemotherapy, this was still recommended for patients with plasma EBV DNA > 0 in the middle of IMRT because of its significantly better association with both PFS and OS. Thus, for patients with NPC with plasma EBV DNA levels > 0 copies/mL in the middle of IMRT, 200 mg/m^2^ DDP is not a recommendable strategy.

ChemoRT, including concurrent DDP chemoradiotherapy, is widely used to treat locoregionally advanced NPC. Lee et al^14^^,^^15^ reported in NPC-9901 and NPC-9902 clinical joint trials that for 100, 200, and 300 mg/m^2^ DDP with concurrent chemoRT, the 5-year locoregional recurrence survival rates of patients with NPC were 79%, 88%, and 88%, respectively, whereas the 5-year DMFS was 68%, 78%, and 77%, respectively. Additionally, Chan et al^21^ reported that more than 2 courses of concurrent chemoRT may not be beneficial to patients. The results of a retrospective study^22^ conducted at the XXXX was also in accordance with those of the previously mentioned studies conducted in Hong Kong. One possible reason for these results was that the therapeutic decisions made in these studies were mainly based on the TNM staging system and did not include EBV DNA levels as a risk factor.

Recently, studies have shown that plasma EBV DNA level combined with TNM staging can screen high-risk patients with NPC.^2^^,^^17^^,^^23^ The TNM stage reflects the anatomic range of the tumor, and because of the heterogeneity of NPC, the prognosis of patients with the same tumor stage can be significantly different in patients with different plasma EBV DNA levels

In our cohort, 1278 patients (67.9%) had plasma EBV DNA level = 0 copies/mL in the middle of IMRT and only 603 (47.2%) had plasma EBV DNA levels > 0 copies/mL in the middle of IMRT. This indicated that approximately two-thirds of the patients with NPC might benefit from concurrent chemotherapy with 200 mg/m^2^ cisplatin. However, the results may be hypothesis generating rather than confirmatory because 200 mg/m^2^ DDP only had similar OS rates compared with >200 mg/m^2^. This finding is consistent with that of a previous study, which focused on an appropriate dose of DDP during IMRT.^8^^,^^22^ However, according to our cohort, one-third of the patients with NPC who showed plasma EBV DNA levels > 0 copies/mL in the middle of IMRT showed better PFS and OS with the administration of >200 mg/m^2^ DDP. In this case, the accompanying incremental side effects were acceptable as the improvements in PFS and OS were significant. Hence, >200 mg/m^2^ DDP for concurrent chemotherapy may be recommended for patients with plasma EBV DNA levels > 0 copies/mL in the middle of IMRT. The previously mentioned EBV DNA-based risk classification in the middle of IMRT could provide timely advice on the initiation of the third cycle of DDP chemotherapy.

Our study has several limitations. First, this was a retrospective study conducted at a single cancer center; therefore, our results must be validated using other data sets and prospective studies. Second, the lack of quality-of-life data for the different treatment methods makes these results underpowered. Thus, a well-designed, multicenter, prospective, and randomized study is needed in the future to validate our findings.

## Conclusion

Our study demonstrated that plasma EBV DNA level > 0 copies/mL in the middle of IMRT is a high-risk factor for patients with stage III-IVa NPC. Furthermore, >200 mg/m² DDP for CCRT may be recommended for patients with plasma EBV DNA level > 0 copies/mL in the middle of IMRT, although it would result in more toxic effects; however, this dose may not be recommended for patients with plasma EBV DNA level = 0 copies/mL in the middle of IMRT because >200 mg/m² DDP had similar OS rates compared with the >200 mg/m² DDP group. Considering that the >200 mg/m² DDP group had higher PFS rates in patients with plasma EBV DNA level = 0 copies/mL in the middle of IMRT, the previously discussed results may be hypothesis generating rather than confirmatory. Thus, the results of this study might widen the choice of concurrent chemotherapy that is offered to patients with NPC based on plasma EBV DNA levels in the middle of IMRT. Further prospective randomized clinical trials are necessary to confirm this hypothesis.

## Appendix Supplementary materials

Image, application 1

## Footnotes

Supplementary material associated with this article can be found in the online version at doi:10.1016/j.adro.2022.100908.
