# Supplementary material for: The Prognostic Role of Plasma Epstein-Barr Virus DNA Levels in the Middle of Intensity Modulated Radiation Therapy to Guide Cisplatin Dose Recommendation in Concurrent Chemoradiation Therapy in Patients With Locally Advanced Nasopharyngeal Carcinoma: A Large Cohort Study
Source: Adv Radiat Oncol. 2022 Feb 3;7(3):100908. doi: 10.1016/j.adro.2022.100908 (PMC9133362; doi:10.1016/j.adro.2022.100908)
Supplement: Supplementary file 1 [file mmc1.docx]

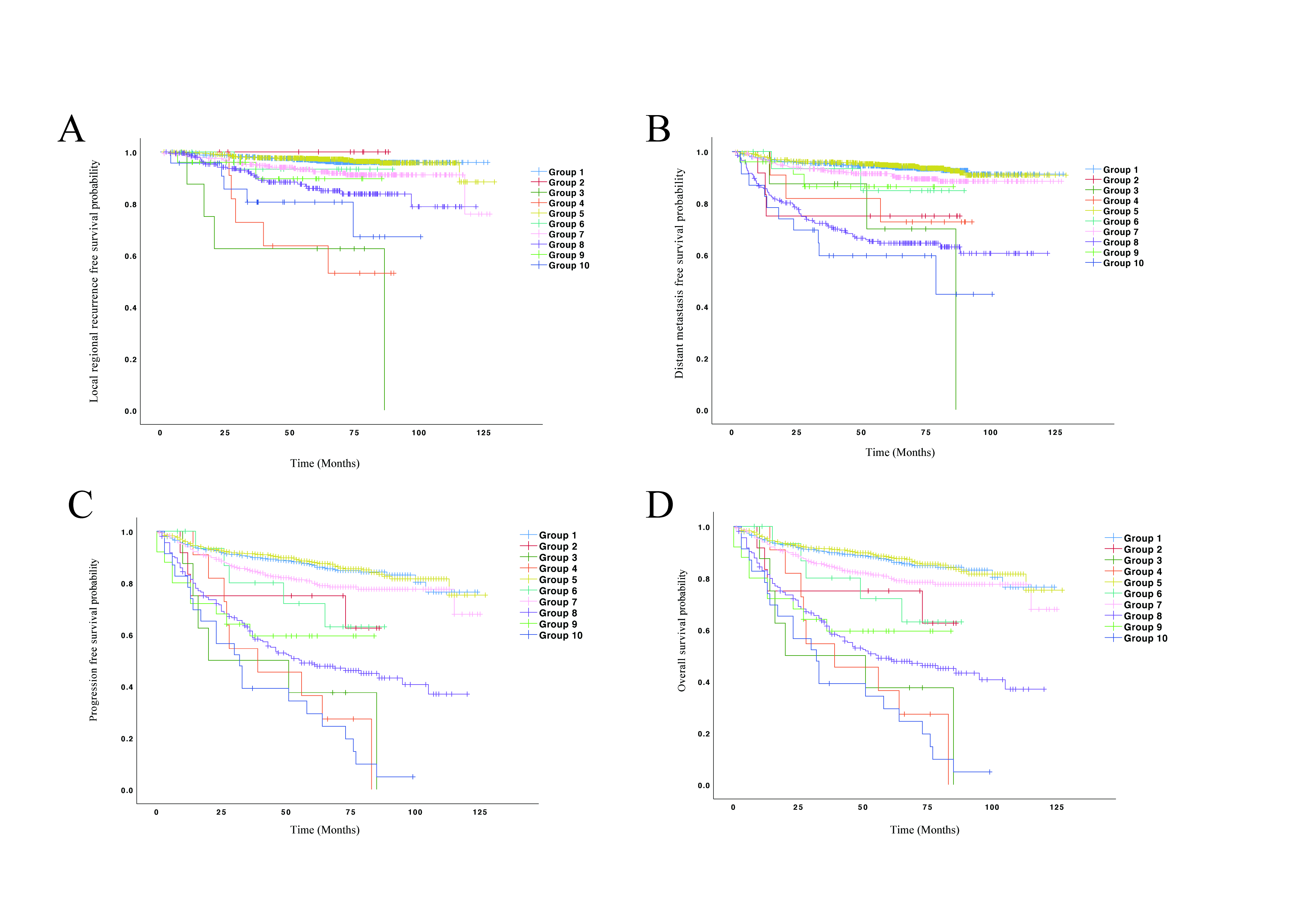


Supplement figure 1 Survival outcomes for patients with different plasma EBV DNA


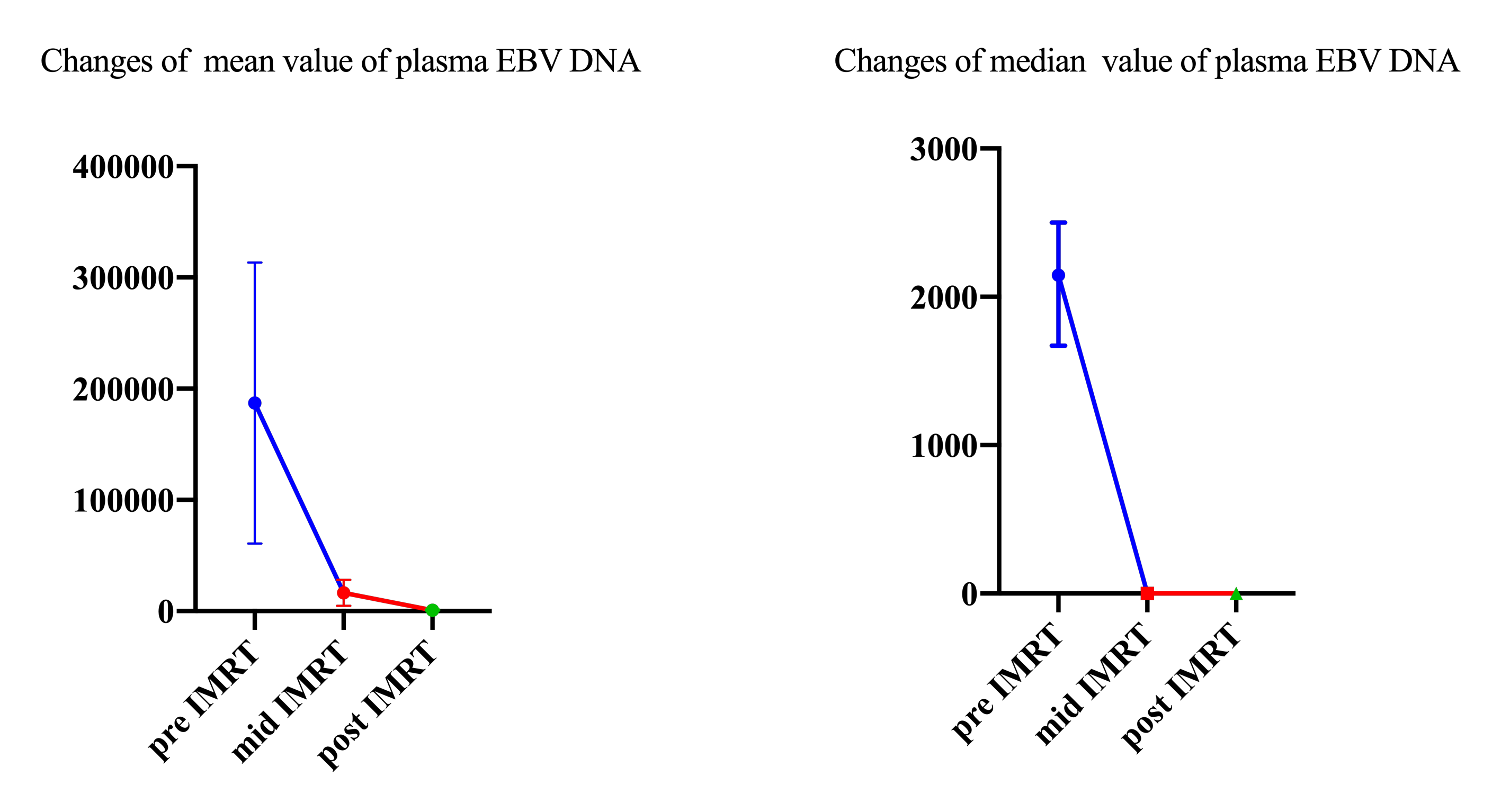


Supplement figure 2 Changes of median and mean value of plasma EBV DNA level during the IMRT.

Supplement table 1. The Survival outcomes for patients in the cohort

| Risk group |  | Overall survival | | Progression free survival | | Distant metastasis free survival | | Local regional recurrence free survival | |
| --- | --- | --- | --- | --- | --- | --- | --- | --- | --- |
| EBV DNA =0 in the middle of IMRT |  | | 95.2 | | 87.1 | | 94.2 | | 97.0 |
| EBV DNA decrease not to 0 in the middle of IMRT |  | | 82.0 | | 67.5 | | 80.6 | | 89.7 |
| EBV DNA increase in the middle of IMRT |  | | 62.5 | | 35.9 | | 71.1 | | 74.2 |
| Merged group | cisplatin | |  | |  | |  | |  |
| EBV DNA =0 in the middle of IMRT | 200 mg/m2 | | 94.9 | | 81.5 | | 86.1 | | 95.6 |
|  | ＞200 mg/m2 | | 94.4 | | 87.6 | | 88.9 | | 96.8 |
| EBV DNA＞0 in the middle of IMRT | 200 mg/m2 | | 73.9 | | 54.8 | | 62.4 | | 82.1 |
|  | ＞200 mg/m2 | | 84.6 | | 72.3 | | 76.4 | | 92.3 |

Supplement table 2. The toxicity of the treatment

| Toxicities  , n (%) | | Total  (grade 1-4)  (n= 1454) | | 200 mg/m2 of cisplatin  (grade 3-4)  (n=474) | Over 200 mg/m2 of cisplatin  (grade 3-4)  (n=980) |
| --- | --- | --- | --- | --- | --- |
| Leucopenia | 1240 (85.3) | | 44 (9.3) | | 169 (17.2) |
| Neutropenia | 1214 (83.5) | | 42 (8.9) | | 153 (15.6) |
| Anemia | 944 (64.9) | | 7 (1.5) | | 15 (1.5） |
| Thrombocytopenia | 255 (17.5) | | 22 (4.6) | | 78 (8.0) |
| ALT increased | 104 (7.2) | | 12 (2.5) | | 43 (4.4) |
| AST increased | 113 (7.8) | | 35 (7.4) | | 81 (8.3) |
| Total protein | 69 (4.7) | | 11 (2.3) | | 30 (3.1) |
| Creatinine increase | 154 (10.6) | | 28 (5.9) | | 88 (9.0) |
| Vomiting | 1378 (94.8) | | 223 (47.0) | | 734 (74.9) |
| Nausea | 1189 (81.8) | | 255 (53.8) | | 765 (78.1) |
| Mucositis | 921 (63.3) | | 143 (30.2) | | 382 (39.0) |
| Dermatitis | 1122 (77.2) | | 19 (4.0) | | 72 (7.3) |
| Hypokalemia | 155 (10.7) | | 21 (4.4) | | 77 (7.9) |
| Hyponatremia | 134 (9.2) | | 23 (4.9) | | 79 (8.1) |
| Late toxicities |  | |  | |  |
| Hearing loss | 332 (22.8) | | 20 (4.2) | | 65 (6.6) |
| Trismus | 55 (3.8) | | 2 (0.4) | | 7 (0.7) |
| Dysphagia | 59 (4.1) | | 7 (1.5) | | 12 (1.2) |
| Neck fibrosis | 303 (20.8) | | 10 (2.1) | | 22 (2.2) |
| Xerostomia | 446 (30.7) | | 21 (4.4) | | 70 (7.1) |
| Cranial nerve palsy | 49 (3.4) | | 0 (0.0) | | 5 (0.5) |
| Radiation encephalopathy | 66 (4.5) | | 3 (0.6) | | 11 (1.1) |
